# 17-DMAG diminishes hemorrhage-induced small intestine injury by elevating Bcl-2 protein and inhibiting iNOS pathway, TNF-α increase, and caspase-3 activation

**DOI:** 10.1186/2045-3701-1-21

**Published:** 2011-06-03

**Authors:** Juliann G Kiang, Neil G Agravante, Joan T Smith, Phillip D Bowman

**Affiliations:** 1 Radiation Combined Injury Program, Armed Forces Radiobiology Research Institute, Uniformed Services University, Bethesda, Maryland, USA; 2 Department of Radiation Biology, Uniformed Services University, Bethesda, Maryland, USA; 3 Department of Medicine, Uniformed Services University, Bethesda, Maryland, USA; 4 US Army Institute of Surgical Research, Fort Sam Houston, Texas, USA

## Abstract

**Background:**

Hemorrhage increases inducible nitric oxide synthase (iNOS) and depletes ATP levels in various tissues. Previous studies have shown that geldanamycin, an inducer of heat shock protein 70kDa (HSP-70) and inhibitor of iNOS, limits both processes. Reduction in NO production limits lipid peroxidation, apoptosome formation, and caspase-3 activation, thereby increasing cellular survival and reducing the sequelae of hemorrhage. The poor solubility of geldanamycin in aqueous solutions, however, limits its effectiveness as a drug. 17-DMAG is a water-soluble analog of geldanamycin that might have greater therapeutic utility. This study investigated the effectiveness of 17-DMAG at reducing hemorrhagic injury in mouse small intestine.

**Results:**

In mice, the hemorrhage-induced iNOS increase correlated with increases in Kruppel-like factor 6 (KLF6) and NF-kB and a decrease in KLF4. As a result, increases in NO production and lipid peroxidation occurred. Moreover, hemorrhage also resulted in decreased Bcl-2 and increased TNF-α, IL-6, and IL-10 concentrations, p53 protein, caspase-3 activation, and cellular ATP depletion. A shortening and widening of villi in the small intestine was also observed. Treatment with 17-DMAG significantly reduced the hemorrhage-induced increases in iNOS protein, jejunal alteration, and TNF-α and IL-10 concentrations, but 17-DMAG did not affect the hemorrhage-induced increases in p53 and IL-6 concentration. 17-DMAG treatment by itself upregulated HSP-70, Bcl-2, and p53.

**Conclusion:**

Since 17-DMAG is water soluble, bioactive, and not toxic, 17-DMAG may prove useful as a prophylactic drug for hemorrhage.

## Background

Hemorrhagic shock has been shown to cause systemic inflammation response syndrome (SIRS), multiple organ dysfunction syndrome (MODS), and multiple organ failure (MOF) [1]. Tissue hypoxia resulting from hemorrhage causes acute increases in intracellular free calcium, 5-lipoxygenase, lipid peroxidation, cyclooxygenase (COX), constitutive nitric oxide synthase, leukotriene B4, prostaglandin E2, interleukins, tumor necrosis factor-α (TNF- α), caspases, Kruppel-like factor 6 (KLF6), inducible nitric oxide synthase (iNOS) [2]. There are also delayed increases in heat shock protein 70 kDa (HSP-70) and hypoxia-inducible factor-1α (HIF1α) [3]. Hemorrhage upregulates iNOS as a result of increases in c-jun, KLF6, and NF-kB and a decrease in KLF4 [4,5]. Increased iNOS elevates NO production, protein nitration, lipid peroxidation, apoptosome formation, and caspase activation; thereby leading to caspase-dependent apoptosis [4,5].

iNOS gene silencing [5,6] or treatment with iNOS inhibitors such as androstenediol [7], L-NAME, or L-NIL-6 [5] reduces hemorrhage-induced injuries. In our laboratory we have shown that geldanamycin, a natural product from the bacterium Streptomyces hygroscopicus that binds with high affinity to the ATP binding pocket of HSP-90, protects mice [4] and rats [8] from hemorrhage-associated organ damage. A single subcutaneous injection of geldanamycin (1 μg/g in 10% DMSO-saline solution) effectively inhibited iNOS induction and activation, NO production, lipid peroxidation, caspase-3 activation, and apoptosis [3,4].

**Figure 1:**
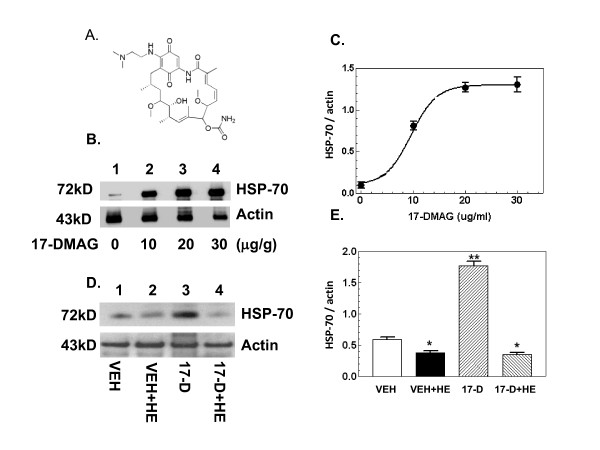
**17-DMAG treatment increases HSP-70**. (A) 17-DMAG structure. (B) Mice were treated with various doses of 17-DMAG 16 hr prior to sham hemorrhage. After sham hemorrhage mice were allowed to respond for 6 hr before sample collection. Representative gel shown. (C) Dose-response curve for various doses of 17-DMAG and HSP-70 (n = 5 per group). Estimated median effective dose is 10 ± 1 μg/g, calculated by Prism program 3.0. (D) Mice were treated with vehicle or 17-DMAG at 10 mg/kg 16 hr prior to hemorrhage. After hemorrhaged mice and sham mice were allowed to respond for 6 hr before sample collection. (n = 5 per group). Representative gel shown. (E) Densitometric quantitation of HSP70 protein bands in panel C. *P < 0.05 vs. VEH and 17-D; **P < 0.05 vs. VEH, VEH+HE, and 17-D+HE. VEH: vehicle; HE: hemorrhage; 17-D: 17-DMAG

Geldanamycin has several drawbacks as a drug, however. Its potential toxicity, metabolic instability, poor solubility in aqueous solutions, and the requirement that it be administered by injection instead of orally limit its effectiveness. 17-DMAG [17-(dimethylaminoethylamino)-17-demethoxygeldanamycin; Figure 1A], a water-soluble, bioactive analogue of geldanamycin [9,10], would appear to have greater utility. Oral effectiveness would make it especially useful in mass-casualty situations. An advantage that 17-DMAG has over a number of other iNOS inhibitors is that it induces potentially cytoprotective HSP-70 protein levels [11]. It is known that increased levels of HSP-70 can protect cells from tissue injury caused by a variety of stressful stimuli, including trauma and disease [12], by inhibiting iNOS and thereby preventing apoptosis and autophagy.

In this investigation, we explored the potential of 17-DMAG to reduce hemorrhage injury. We used a small intestine model for this study because it is an organ especially sensitive to hemorrhage injury, responding within 1 hr [3,4]. It is known that hemorrhage increases iNOS-mediated caspase-3 cellular activity in various tissues [3,4,6,13] and that caspase-3 plays a key role in activating many downstream caspases involved in apoptosis [14,15]. We are the first to report that 17-DMAG limits hemorrhage-induced injury in small intestine *in vivo *by elevating Bcl-2 protein and inhibiting the iNOS pathway, TNF-α increases, and caspase-3 activation.

## Results

### 17-DMAG treatment increases inducible HSP-70

Because geldanamycin treatment induces HSP-70 [4,8], we sought to determine if 17-DMAG would also do so. Mice were orally administered with either vehicle or various doses of 17-DMAG 16 hr prior to euthanization and tissue collection. Western blot analysis showed 17-DMAG increased HSP-70 in a dose-dependent manner, with a medium effective dose of 10 ± 1 μg/g (Figure 1B-C). Hemorrhage alone significantly inhibited HSP-70 (Figure 1D, lane 2 vs. lane 1 and Figure 1E). Treatment with 17-DMAG followed by hemorrhage resulted in lower HSP-70 levels than after treatment with 17-DMAG alone, and the levels observed were not significantly different from levels observed after hemorrhage alone (Figure 1D, lane 4 vs. lane 3 and Figure 1E).

### 17-DMAG treatment inhibits hemorrhage-induced increases in iNOS and KLF6 and decrease in KLF4

**Figure 2:**
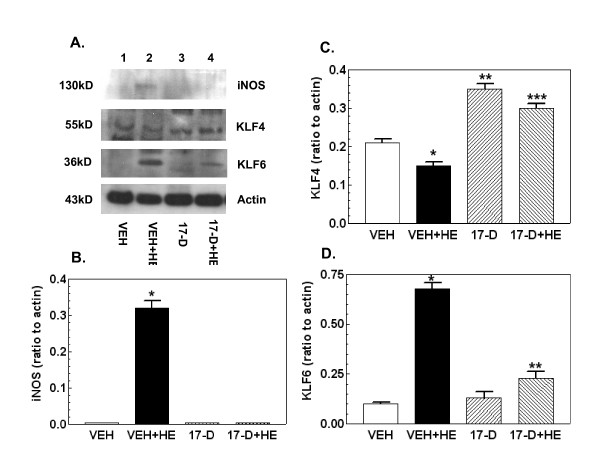
**17-DMAG treatment inhibits hemorrhage-induced increases in iNOS and KLF6 and decrease in KLF4**. Mice were treated with 10 mg/kg 17-DMAG 16 hr prior to hemorrhage. After hemorrhage mice were allowed to respond for 6 hr before sample collection (n = 4-5 per group). (A) Representative gel shown. (B) Densitometric quantitation of iNOS protein bands in panel A. *P < 0.05 vs. VEH, 17-D, and 17-D+HE. (C) Densitometric quantitation of KLF4 protein bands in panel A. *P < 0.05 vs. VEH, 17-D, and 17-D+HE; **P < 0.05 vs. VEH, VEH+HE, and 17-D+HE; ***P < 0.05 vs. VEH, VEH+HE, and 17-D. (D) Densitometric quantitation of KLF6 protein bands in panel A. *P < 0.05 vs. VEH, 17-D, and 17-D+HE; **P < 0.05 vs. VEH, VEH+HE, and 17-D. VEH: vehicle; HE: hemorrhage; 17-D: 17-DMAG

Consistent with previous reports [3-5,7], Figure 2 shows that hemorrhage increased iNOS and KLF6 and decreased KLF4 (lane 2 vs. lane 1). 17-DMAG treatment prior to hemorrhage reversed these changes (lane 4 vs. lane 2). 17-DMAG treatment alone did not change the basal levels of iNOS and KLF6 but increased the KLF4 baseline (lane 3 vs. lane 1).

### 17-DMAG treatment inhibits hemorrhage-induced NF-κB

**Figure 3:**
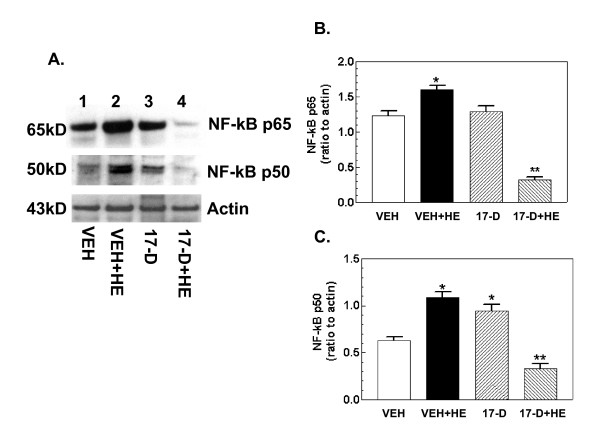
**17-DMAG treatment inhibits hemorrhage-induced increases in NF-κB**. Mice were treated with 10 mg/kg 17-DMAG 16 hr prior to hemorrhage. After hemorrhage mice were allowed to respond for 6 hr before sample collection (n = 4-5 per group). (A) Representative gel shown. (B) Densitometric quantitation of NF-κB p65 protein bands in panel A. *P < 0.05 vs. VEH, 17-D, and 17-D+HE; **P < 0.05 vs. VEH, VEH+HE, and 17-D. (C) Densitometric quantitation of NF-κB p50 protein bands in panel A. *P < 0.05 vs. VEH and 17-D+HE. VEH: vehicle; HE: hemorrhage; 17-D: 17-DMAG

The hemorrhage-induced increase in iNOS could be a result of increases in NF-κB since the promoter region of the iNOS gene contains 10 NF-κB binding motifs [3]. We, therefore, evaluated levels of the NF-κB p65 and p50 transcription factors after hemorrhage. Figure 3 shows that hemorrhage increased NF-κB p65 and p50 (lane 2 vs. lane 1). 17-DMAG treatment prior to hemorrhage inhibited the increases (lane 4 vs. lane 2). 17-DMAG treatment alone did not change the NF-κB p65 basal levels but increased the p50 baseline (lane 3 vs. lane 1).

### 17-DMAG treatment inhibits hemorrhage-induced increases in NO and lipid peroxidation and decrease in cellular ATP depletion

**Figure 4:**
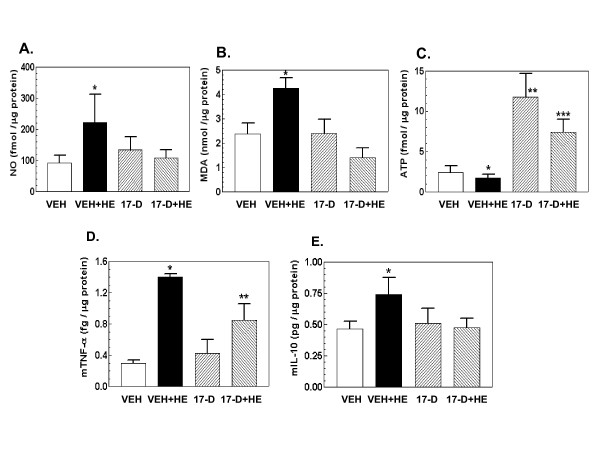
**17-DMAG treatment inhibits hemorrhage-induced increases in NO, lipid peroxidation, ATP depletion, and cytokines**. Mice were treated with 10 mg/kg 17-DMAG 16 hr prior to hemorrhage. After hemorrhage mice were allowed to respond for 6 hr before sample collection (n = 4-5 per group). Panels indicate levels measured as described in Methods: NO (A), MDA (B), cellular ATP (C), TNF-α (D), and IL-10 (E). For panels A, B, and E: *P < 0.05 vs. VEH, 17-D, and 17-D+HE. For panel C: *P < 0.05 vs. VEH, 17-D, and 17-D+HE; **P < 0.05 vs. VEH, VEH+HE, 17-D+HE; ***P < 0.05 vs. VEH, VEH+HE, and 17-D. VEH: vehicle; HE: hemorrhage; 17-D: 17-DMAG

In agreement with previous reports [4,7], hemorrhage increased NO production and lipid peroxidation as indicated by malondialdehyde (MDA) production and cellular ATP depletion (Figure 4A-C). 17-DMAG treatment prior to hemorrhage inhibited these increases. 17-DMAG treatment alone did not alter NO and MDA basal levels but increased the cellular ATP baseline (Figure 4A-C).

### 17-DMAG treatment inhibits hemorrhage-induced increases in TNF-α and IL-10

Lad et al. [16] showed that hemorrhage increases TNF-α, IL-8, and other cytokines. Using ELISA, we found that hemorrhage increased TNF-α (Figure 4D), IL-10 production (Figure 4E), and IL-6 (data not shown). 17-DMAG treatment prior to hemorrhage significantly inhibited the increases in TNF-α (Figure 4D) and IL-10 production (Figure 4E), but not IL-6 (data not shown). 17-DMAG treatment alone did not alter their basal levels (Figure 4D-E).

### 17-DMAG treatment inhibits hemorrhage-induced caspase-3 activation

**Figure 5:**
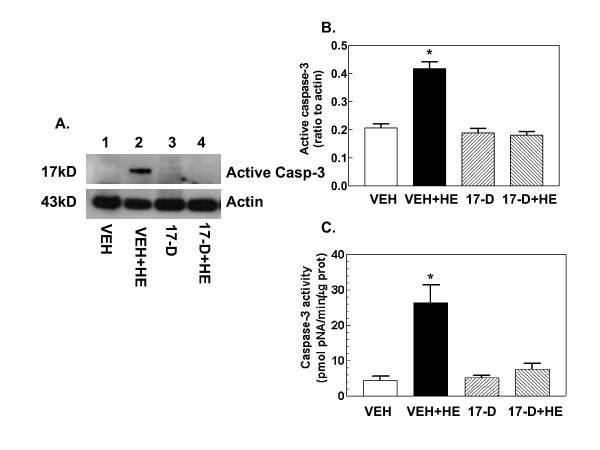
**17-DMAG treatment inhibits hemorrhage-induced caspase-3 activation**. Mice were treated with 10 mg/kg 17-DMAG 16 hr prior to hemorrhage. After hemorrhage mice were allowed to respond for 6 hr before sample collection (n = 4-5 per group). (A) Representative gel shown. (B) Densitometric quantitation of active caspase-3 protein bands in panel A. *P < 0.05 vs. VEH, 17-D, and 17-D+HE. (C) Corresponding caspase-3 enzymatic activity. *P < 0.05 vs. VEH, 17-D, and 17-D+HE. VEH: vehicle; HE: hemorrhage; 17-D: 17-DMAG

Hemorrhage activated caspase-3 enzymatic activity (Figure 5A) and increased levels of the active form of caspase-3 protein (Figure 5B, lane 2 vs. lane 1). 17-DMAG treatment prior to hemorrhage fully inhibited caspase-3 enzymatic activity and its protein production (Figure 5A-C). 17-DMAG treatment alone did not change the basal levels.

### 17-DMAG treatment increases p53 and Bcl-2

**Figure 6:**
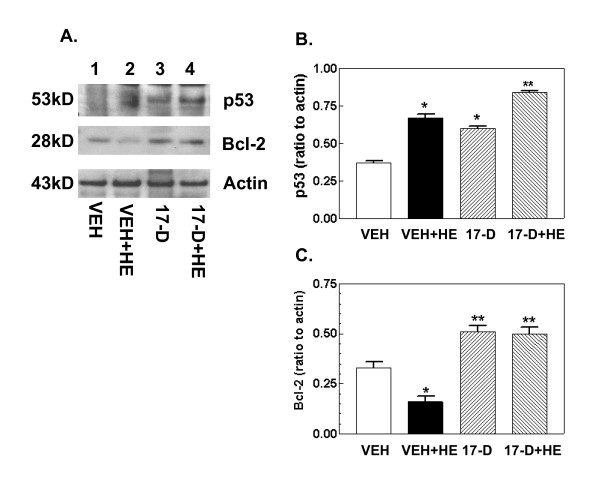
**17-DMAG treatment increases p53 and Bcl-2**. Mice were treated with 10 mg/kg 17-DMAG 16 hr prior to hemorrhage. After hemorrhage mice were allowed to respond for 6 hr before sample collection (n = 4-5 per group). (A) Representative gel shown. (B) Densitometric quantitation of p53 protein bands in panel A. *P < 0.05 vs. VEH and 17-D+HE; **P < 0.05 vs. VEH, VEH+HE, and 17-D. (C) Densitometric quantitation of Bcl-2 protein bands in panel A. *P < 0.05 vs. VEH, 17-D, and 17-D+HE; **P < 0.05 vs. VEH and VEH+HE. VEH: vehicle; HE: hemorrhage; 17-D: 17-DMAG

It is known that p53 is pro-apoptotic, whereas Bcl-2 is anti-apoptotic [3,17]. To determine if caspase-3 activation was mediated by the p53 pathway, p53 and Bcl-2 were measured by Western blotting. Figure 6 shows that hemorrhage increased p53 and decreased Bcl-2 (Figure 6A, lane 2 vs. lane 1). When 17-DMAG was administered prior to hemorrhage, jejunum displayed increased levels of both p53 and Bcl-2 (lane 4 vs. lanes 2 and 1). 17-DMAG treatment alone also increased both p53 and Bcl-2 (lane 3 vs. lane 1).

### 17-DMAG treatment inhibits hemorrhage-induced injury in jejunum

**Figure 7:**
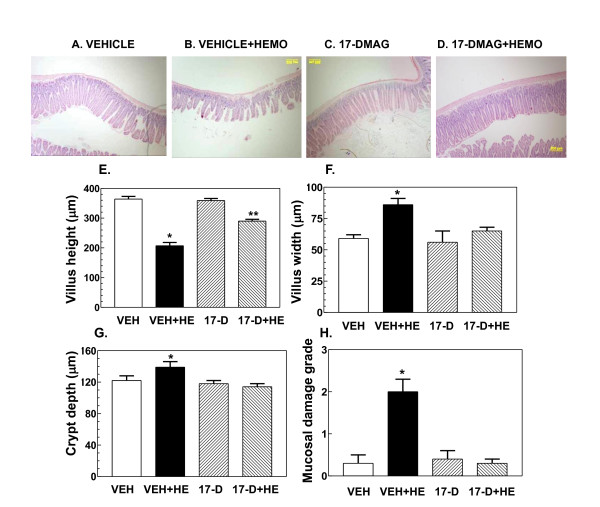
**17-DMAG treatment inhibits hemorrhage-induced injury in jejunum**. Mice were treated with 10 mg/kg 17-DMAG 16 hr prior to hemorrhage. After hemorrhage mice were allowed to respond for 6 hr before sample collection. (n = 4-5 per group). (A-D) Representative histology slides from jejunum of mice treated with VEH, VEH+HEMO, 17-DMAG, or 17-DMAG+HEMO. (E) Villus height measurements after treatments. *P < 0.05 vs. VEH, 17-D, and 17-D+HE; **P < 0.05 vs. VEH, VEH+HE, and 17-D. (F) Villus width measurements after treatments. *P < 0.05 vs. VEH, 17-D, and 17-D+HE. (G) Crypt depth measurements after treatments. *P < 0.05 vs. VEH, 17-D, and 17-D+HE. (H) Mucosal damage assessments. *P < 0.05 vs. VEH, 17-D, and 17-D+HE. VEH: vehicle; HE or HEMO: hemorrhage; 17-D: 17-DMAG

Hemorrhage injured the jejunal structure. Villus appearance changed (Figure 7B vs. 7A); hemorrhage reduced villus height (Figure 7E) and increased villus width (Figure 7F) but did not alter crypt depth (Figure 7G). Mucosal damage, assessed using a six-tier scale [3], indicated level 2.0 ± 0.3 damage for hemorrhage jejunum compared to level 0.3 ± 0.2 for sham jejunum (Figure 7H). 17-DMAG treatment prior to hemorrhage prevented the hemorrhage-induced damage (Figure 7D vs. 7A); villus heights (Figure 7E) and widths (Figure 7F) in treated animals were not significantly different from those of jejunum in sham-treated mice. 17-DMAG treatment alone did not alter jejunum morphology (Figure 7C and 7E-H).

## Discussion

Our data indicate that hemorrhage-induced injuries to small intestine were reduced by pretreatment with orally administered 17-DMAG. The protection we observed correlated with a 17-DMAG-induced reduction in iNOS and caspase-3 activity and an increase in Bcl-2 protein.

Hemorrhage can occur after a wide variety of injuries, ranging from wound trauma to stroke, aneurysm, birth labor, or blast impact. Hemorrhage lowers the levels of oxygen and nutrition available to tissues and results in an accumulation of carbon dioxide and metabolic waste, leading to activation of signal transduction pathways and tissue damage [2]. In our experiments, hemorrhage activated the iNOS pathway (Figures 2 and 3), generated oxidative and nitrosative stress (Figure 4), and reduced cellular ATP (Figure 4). Subsequently, caspase-3 was activated (Figure 5) and cell death occurred (Figure 7). These observations are consistent with those previously described [4-7,17]. The hemorrhage-induced increase in iNOS protein was due to increases in transcription factors NF-κB and KLF6 and a decrease in KLF4 (Figures 2,3). It is known that NF-κB and KLF6 stimulate iNOS gene expression whereas KLF4 inhibits it [3].

17-DMAG treatment inhibited activation of the iNOS pathway and caspase-3-mediated cell death mainly by inhibiting NF-κB and KLF6 and increasing KLF4. The interpretation that iNOS inhibition leads to reduced hemorrhage damage is reinforced by the results of studies employing iNOS gene knockout [17] and treatment with iNOS-specific inhibitors [6,18]. How 17-DMAG exerts its action on these iNOS transcription factors is not completely clear and further study is required.

An increase in HSP-70 has been shown to inhibit the iNOS increase triggered by hemorrhage [4,8]. However, it does not appear that the ability of 17-DMAG to inhibit hemorrhage damage in jejunum is due to its ability to increase HSP-70. 17-DMAG treatment at the EC50 concentration for inducing HSP-70 markedly elevated HSP-70 levels, but the combination of 17-DMAG treatment and hemorrhage resulted in a lower level of HSP-70 that was no different than that seen after hemorrhage alone.

Because 17-DMAG in our experiments was administered orally, the possibility that 17-DMAG was absorbed by small intestine, and locally protected it from hemorrhage injury cannot be excluded. It is known that hemorrhage induces a p38-MAPK increase [19] and a Bcl-2 decrease (Figure 6) [19]. 17-DMAG may exert an effect on these proteins as well. More studies are needed in this area.

Hemorrhage increased ATP depletion in agreement with our previous report [20]. 17-DMAG treatment prevented the depletion, probably due to its ability to upregulate production of pyruvate dehydrogenase protein [20].

Hemorrhage increased TNF-α and IL-10 (Figure 4D-E) and IL-6. Increased TNF-α has been observed in other experimental models such as those for ionizing radiation [21-25] and ischemia [26]. Inhibition of TNF-α levels by drug treatment or antibody neutralization results in an improvement in bone marrow regeneration after radiation exposure [25]. Yen et al. [27] reported that TNF-α is the key to the development of hemorrhage injury; Pfeifer et al. [28] showed that hemorrhage in fact triggers increased TNF-α. It is likely in our experiments that apoptosis was also caused by the extrinsic pathway involving TNF-α receptors, which probably contributed to the final caspase-3 activation. The fact that 17-DMAG inhibited the hemorrhage-induced increase in TNF-α is desirable, but the fact it also inhibited the beneficial IL-10 is undesirable in terms of 17-DMAG's use as a drug. The hemorrhage-induced increase in IL-10 we observed could be a self-defense response to injury, but its level is in the range of pg/ml, a level too low to combat the damage. We have shown that treatment with ng/ml levels of IL-10 are required to improve survival from a lethal dose of γ-irradiation [29].

Bcl-2 is anti-apoptotic and p53 is pro-apoptotic [30]. Hemorrhage reduced Bcl-2 and increased p53 in our experiments, which probably partly contributed to the damage observed. However, 17-DMAG treatment increased not only Bcl-2 but surprisingly also p53 (Figure 6). The anti-apoptotic activity of Bcl-2 stimulated by 17-DMAG is apparently greater than the pro-apoptotic activity of p53.

To summarize, 17-DMAG is water-soluble, distributes to all tissues in the body, and is non-toxic in mice [9,10]. Compared to other iNOS inhibitors, 17-DMAG is potentially superior because of its ability to increase HSP-70 and Bcl-2 proteins and decrease HSP-90 activity and TNF-α concentration as well as ATP depletion. Its water solubility and non-toxicity and the fact that 17-DMAG is effective when administered orally make it a promising candidate drug for ameliorating hemorrhage injury.

## Conclusion

Hemorrhage reduced the basal levels of HSP-70 and Bcl-2, increased iNOS protein and its transcription factors, elevated NO production, lipid peroxidation, ATP depletion, TNF-α, IL-6, IL-10 concentrations, caspase-3 activation, p53, and jejunum damage. Oral treatment with 17-DMAG increased Bcl-2 and inactivated the caspase pathway by inhibiting the iNOS pathway and the TNF-α increase so as to diminish jejunum injury despite the fact it did not reduce p53. These results suggest that 17-DMAG may have significant value in preventing tissue damage associated with hemorrhage.

## Methods

### Hemorrhage

Animal protocols were reviewed and approved by Institutional Animal Care and Use Committee at US Army Institute of Surgical Research. Experiments were conducted using the mouse model described by Song et al. [31]. Male Swiss Webster mice weighing 25-35 g were anesthetized with 5% isoflurane/oxygen (EZ Systems, Palmer, PA) and 40% of their calculated blood volume [32] removed by cardiac puncture over a period of 1 minute using a 26 gauge needle and 1.0 ml syringe essentially as previously described [33]. Sham animals were anesthetized and the needle inserted into the heart but no blood was withdrawn. This procedure took no more than 3 min to perform. Mean arterial blood pressure fell from 80 to 40 mmHg within 2 h after treatment [31]. Mice were then allowed to respond to the treatments for an additional 6 hr before they were euthanized. A small section of jejunum was then collected for histopathology assessment, immunoblotting analysis, and biochemical assays. Small intestine was chosen for investigation in this study because it is a sensitive organ acutely damaged by hemorrhage [3,4].

### 17-DMAG treatment

17-DMAG [17-(dimethylaminoethylamino)-17-demethoxygeldanamycin] (CAS Registry No.: 707545) was obtained from InvivoGen (San Diego, CA) and dissolved in 0.2 ml 5% dextrose saline for use. 17-DMAG is an analog of geldanamycin, a natural product produced by Streptomyces hygroscopicus that binds with high affinity to the ATP binding pocket of HSP-90 [9,10]. 17-DMAG is water soluble, less metabolizable than geldanamycin, and distributes to all tissues (http://clinicaltrials.gov/ct/show/NCT00248521). 17-DMAG (or dextrose-saline vehicle) was administered at various doses to mice orally using feeding needles 16 h prior to hemorrhage.

### Histopathologic assessment

Jejunal tissue specimens were rinsed in cold saline solution and immediately fixed in 10% buffered formalin phosphate. The tissue was then embedded in paraffin, sectioned transversely, and stained with H&E. The mucosal crypt depth and width and villus height were measured. Overall jejunal mucosal damage for each specimen was graded using a six-tiered scale [3].

### Western blots

Jejunal tissue was minced in 100 μl Hanks' balanced salt solution (HBSS), sonicated, and centrifuged at 12,500 x g for 10 min. The supernatant was then collected. The total protein in the jejunal lysate was determined with Bio-Rad reagent (Bio-Rad Laboratories, Richmond, CA, USA). To investigate synthesis of HSP-70, iNOS, iNOS transcription factors, actin, Bcl-2, and caspase-3, aliquots containing 20 μg of protein in tris buffer (pH = 6.8) containing 1% sodium dodecyl sulfate (SDS) and 1% 2-mercaptoethanol were resolved on SDS-polyacrylamide slab gels (Novex precast 4-20% gel; Invitrogen, Carlsbad, CA, USA). After electrophoresis, proteins were blotted onto a PDVF membrane (type NC, 0.45 μm; Invitrogen) using a Novex blotting apparatus and the manufacturer's protocol. After blocking the membrane by incubation in tris-buffered saline-0.5% tween20 (TBST) containing 3% nonfat dried milk for 90 min at room temperature, the blot was incubated for 60 min at room temperature with monoclonal antibodies directed against actin, KLF4, KLF6, NF-kB p65, NF-kB p50, HSP70, Bcl-2 (Santa Cruz Biotechnology, Santa Cruz, CA, USA), iNOS (BD Signal Transduction, Sparks, MD, USA), and caspase-3 (Epitomics, Burlingame, CA, USA) at a concentration of 1 μg/ml in TBST - 3% dry milk. The blot was then washed 3 times (10 min each) with TBST before incubating the blot for 60 min at room temperature with a 1000X dilution of species-specific IgG peroxidase conjugate (Santa Cruz Biotechnology) in TBST. The blot was washed 6 times (5 min each) in TBST before detection of peroxidase activity using the Enhanced Chemiluminescence Plus (Amersham Life Science Inc., Arlington Heights, IL, USA). Actin levels were not altered by hemorrhage; we therefore used actin as a control for protein loading. Protein bands of interest were quantified densitometrically and normalized to actin [4].

### Nitric oxide measurements

NO production was measured under acidic conditions as nitrite, using a commercial kit (Biomedical Research Service, School of Medicine and Biomedical Sciences, State University of New York at Buffalo, NY, USA, http://www.bmrservice.com).

### Lipid peroxidation measurements

Malondialdehyde (MDA), a lipid peroxidation end product, was measured colorimetrically using a commercial lipid peroxidation assay kit (CalBiochem, San Diego, CA, USA).

### Cellular ATP measurements

Cellular ATP levels were determined using the ATP Bioluminescence Assay Kit HS II (Roche, Mannheim, Germany). Luminescence was measured with TD-20/20 luminometer (Turner Designs, Sunnyvale, CA, USA). Data were normalized to total protein and the cellular ATP level was expressed as fmol/μg protein.

### Cytokine measurements

IL-6, IL-10, and TNF-α in jejunal lysates were measured using an ELISA kit (Biocompare, South San Francisco, CA, USA) according to the manufacturer's protocol. The data are presented as pg/ml.

### Caspase-3 enzymatic activity measurements

Caspase-3 activity was determined using the CASPASE-3 Cellular Activity Assay Kit PLUS (Biomol, Plymouth Meeting, PA, USA). In brief, 10 μl of each sample lysate was added to wells of a 96-well plate already containing the substrate Ac-DEVD-pNitroaniliane (Ac-DEVD-pNA). Caspase in the lysates cleaved the substrate to pNA as indicated by an increase in absorbance at 405 nm, which was used to determine reaction rate. Absorbance was measured using a SpectraMax 250 spectrophotometric plate reader and SOFTmax Pro 3.1.1 software (Molecular Devices, San Diego, CA, USA). Data were normalized to total protein, and caspase-3 activity was expressed as pmol pNA/min/μg protein.

### Solutions

Hanks' balanced salt solution contained in mM: 145 NaCl, 4.5 KCl, 1.3 MgCl2, 1.6 CaCl2, and 10 HEPES (pH 7.40 at 24°C).

### Statistical analysis

All data are expressed as mean ± S.E.M. One-way ANOVA, Studentized-range test, and Bonferroni inequality were used for comparison of groups with 5% as a significant level.

### Abbreviations

17-DMAG - 17-(dimethylaminoethylamino)-17-demethoxygeldanamycin; iNOS - inducible nitric oxide synthase; SIRS - systemic inflammation response syndrome; MODS - multiple organ dysfunction syndrome; MOF - multiple organs failure; TNF-α - tumor necrosis factor-α; KLF - Kruppel-like factor; NO - nitric oxide; MDA - malondialdehyde; IL - interleukin; HIF1α - hypoxia-inducible factor-1α; NF-κB - nuclear factor- κB; HSP-70 - heat shock protein 70 kDa; HSP-90 - heat shock protein 90 kDa; VEH: vehicle; HE - hemorrhage; 17-D - 17-DMAG.

## Competing interests

The authors declare that they have no competing interests.

## Authors' contributions

JGK and PDB conceived of the study and participated in its design, execution, and preparation of the manuscript. PDB performed hemorrhage, tissue collection, and histopathology. JGK, NGA, and JTS performed immunoblotting, biochemical assays, data collection, and data analysis. All authors read and approved the final manuscript.
